# Use of Rimegepant in the Management of Cervicogenic Headache Secondary to Trauma: A Case Series

**DOI:** 10.7759/cureus.34662

**Published:** 2023-02-05

**Authors:** Shin T Zaw, Thinzar Zaw, Benito Torres

**Affiliations:** 1 Medicine, Lake Erie College of Osteopathic Medicine, Bradenton, USA; 2 Medicine, University of Central Florida College of Medicine, Orlando, USA; 3 Pain Management, Bartow Regional Medical Center, Lakeland, USA

**Keywords:** cervicalgia, migraine, headache, pain management, trauma

## Abstract

A cervicogenic headache (CH) originates from a cervical source. Multiple diagnostic criteria and treatment strategies for CH are present. Rimegepant is a calcitonin gene-related peptide receptor (CGRP) antagonist. We present a case series of three patients with CH who reported varying degrees of decreased headache intensity after using rimegepant.

## Introduction

Headaches originating from a cervical source are called cervicogenic headaches (CHs). It occurs in about 2.5% of the general population [1]. The most common source of this headache is the C2-C3 joint [2]. Various interventional and manual treatment strategies are present to treat this headache [3,4]. Rimegepant is a calcitonin gene-related peptide receptor (CGRP) antagonist [5]. In the cases we present, patients report a decrease in the intensity of headaches after using rimegepant. However, there is no established link between CGRP and CH in the available literature.

## Case presentation

Case 1

A 43-year-old female presented with neck pain and headaches after a motor vehicle accident (MVA). The pain has been present for the past three weeks. She reported she experienced a whiplash injury. She denied loss of consciousness. The patient was involved in two prior MVAs in 2001. The cervical spine pain is described as sharp, worse during the night, radiating, and associated with headaches. The patient reported that the cervical spine pain and headaches improved with rest. The patient has a past medical history of anxiety disorder. She is currently being treated with chiropractic care, physical therapy, cyclobenzaprine, and ibuprofen without significantly relieving her pain, along with no evidence of medication overuse.

She denied any history of daily headaches before the accident. She stated she has been experiencing headaches every day after the accident, ranging from one to three episodes a day, and they typically begin an hour after waking up and, in total, last over 12 hours. Sometimes she will only experience one episode without any periods of headache freedom. The patient described the headache pain as being located primarily in the occipital and temporal regions bilaterally, but sometimes it is more diffuse and feels like a "tight band." She described the pain as deep pressure, but when severe, she experienced associated symptoms of nausea and photophobia. She rated these headaches, on average, as a 9 out of 10 but can reach 10 out of 10 on a pain scale.

On examination of the cervical spine, she experienced pain with neck rotation to the right, neck extension, neck flexion, and neck rotation to the left. The trapezius was tender to palpation. The patient suffered from a headache that worsened during the examination. An MRI indicated straightening of the standard cervical lordotic spinal curve, C2-C3, C3-C4, C4-C5, and C5-C6 demonstrated a posterocentral disc herniation compressing the thecal sac. The patient was diagnosed with cervicalgia with associated symptoms of headaches.

The patient was provided a pack of four rimegepant 75mg pills and was instructed to take a single oral dose during the onset of a headache or when experiencing a headache but not to exceed 75 mg per 24-hr period. She reported during the two-week follow-up that the medication reduced her headaches to a 4/10 and provided pain relief for three hours. She denied noticing a decrease in headache episodes.

Case 2

A 43-year-old female presented with neck pain and headaches after an MVA. The pain has been present for the past four months and is worse during the day. She reported experiencing a whiplash injury. She denied loss of consciousness. The patient was involved in a prior MVA in June 2021. The cervical spine pain is described as constant, sharp, stabbing, worse at night, and worse during the day and associated with numbness, tingling, and headaches. The patient reported that cervical spine pain and headaches improved with rest. She is being treated with chiropractic adjustments, physical therapy, ibuprofen, and acetaminophen with codeine without significantly relieving her pain, and there is no evidence of medication overuse.

She denied any history of daily headaches before the accident. She stated she has been experiencing headaches every day since the accident, ranging from one to two episodes a day, and they typically begin two hours after waking up and, in total, last over 12 hours. Sometimes she will only experience one episode without any periods of headache freedom. The patient described the headache pain as being bilaterally located in the frontal and occipital regions. She described the pain as constant and throbbing. She experienced associated symptoms of nausea when severe. She rated these headaches, on average, as an 8 out of 10, but they can reach 10 out of 10 on a pain scale.

On examination of the cervical spine, she experienced left and right paraspinal musculature tenderness to palpation. The cervical spine facet loading test was positive to the left, right, and extension. The examination of the shoulder was unremarkable. The patient suffered from a headache that worsened during the examination. An MRI demonstrated a loss of cervical lordosis, a C5-C6 disc bulge with indentation of the thecal sac, and a C6-C7 disc herniation. The patient was diagnosed with cervicalgia with associated symptoms of headaches.

The patient was provided a pack of four rimegepant 75 mg pills and was instructed to take a single oral dose during the onset of a headache or when experiencing a headache but not to exceed 75 mg per 24-hour period. She reported that during a two-week follow-up, the medication reduced her headaches to a 5 out of 10 and provided pain relief for two hours. She denied noticing any decrease in the recurrence of headaches.

Case 3

A 42-year-old female presented with neck pain and headaches after an MVA. The pain has been present for the past three months. She reported experiencing a whiplash injury. She stated that she did lose consciousness. She denied any prior MVAs. The cervical spine pain is described as constant, sharp, worse during the night, worse with sitting, radiating, tingling, and associated with headaches. The patient reported that cervical spine pain and headaches improved with rest. She is being treated with chiropractic adjustments, physical therapy, and cyclobenzaprine without significantly relieving her pain, and there is no evidence of medication overuse.

She denied any history of daily headaches before the accident. She stated she has been experiencing headaches every day since the accident, ranging from one to three episodes a day, and they typically begin two hours after waking up and, in total, last over 12 hours. Sometimes she will only experience one episode without any periods of headache freedom. The patient described the headache pain as being located bilaterally in the occipital regions but intermittently experiencing pain in the unilateral left temporal area. She described the pain as constant and throbbing. She experienced associated symptoms of nausea when severe. She stated her headaches last greater than 12 hours daily. She rated these headaches, on average, as a 7 out of 10, but they can reach 10 out of 10 on a bad day.

On examination of the cervical spine, she experienced left and right paraspinal musculature tenderness to palpation. The cervical spine's range of motion was grossly limited and painful. The trapezius was tender to palpation. The patient suffered from a headache that worsened during the examination. The cervical spine facet loading test was positive to the left and right. An MRI demonstrated C3-4, C4-5, and C5-6 disc bulge compressing the thecal sac. The patient was diagnosed with cervicalgia with associated symptoms of headaches.

The patient was provided a pack of four rimegepant 75 mg pills and was instructed to take a single oral dose during the onset of a headache or when experiencing a headache but not to exceed 75 mg per 24-hour period. She reported that during a two-week follow-up, the medication reduced her headaches to a 5 out of 10 and provided pain relief for two hours. She denied noticing any decrease in the recurrence of headaches.

## Discussion

CH refers to head pain that originates from the cervical spine. CH is primarily diagnosed clinically. CH diagnostic criteria have been proposed by the International Headache Society (IHS). IHS describes it as follows: "Headache or facial pain attributable to disturbance of the skull, neck, eyes, ears, nose, sinuses, teeth, mouth, or other facial or cervical structure" [6]. The prevalence of CH in the general population is 2.5%, whereas the percentage among patients with severe headaches is 17.5% [1]. According to Lord et al., 53% of patients with whiplash injuries fall into this category [7]. For the diagnosis of CH, there are two schools of thought. One holds that CH can be identified based on specific clinical characteristics. In contrast, the other claims that controlled diagnostic blocks can be used to pinpoint the cervical source of CH [8]. Certain clinical features of CH include unilateral headache associated with neck movement or pressure, concomitant pain in the neck, shoulder, and arm, and restricted neck motion [3]. Patients with CH do not exhibit any specific radiological abnormalities that can distinguish them from other headaches [9]. Fluoroscopically guided, controlled diagnostic blocks are used in interventional procedures to identify the cause of CH. According to Dwyer et al., the C2-C3 joint is the primary cause of CH, and about 70% of cases involve it [10]. The C3-C4 joints are also involved in CH pain, according to Copper et al. [11]. Dissecting aneurysms of the vertebral or internal carotid arteries can cause neck pain and headache; it must be kept in mind as a differential diagnosis of CH, as cervical manipulation for CH can aggravate the aneurysm [12].

In a study by Farina et al., transcutaneous electrical nerve stimulation was used to treat CH patients, and 20% of the patients reported a 40-60% decrease in headache index. In comparison, 80% of the patients reported a reduction of at least 60% [13]. A study by Dunning et al. compared upper cervical and upper thoracic spinal mobilization and exercise to upper cervical and upper thoracic spinal manipulation and electrical dry needling for the treatment of CH patients. According to a three-month follow-up [14], the first group reported a higher reduction in headache frequency, intensity, and duration than the other group. According to Chaibi and Russell's systematic analysis of randomized control trials evaluating the effectiveness of manual therapies for CH, spinal manipulative therapy may be a helpful management strategy [4].

Rimegepant is a small-molecule CGRP receptor antagonist. CGRP, a vasodilatory neuropeptide, has a role in the pathogenesis of migraine, a highly disabling neurovascular disorder characterized by violent headache attacks. The FDA has recently approved rimegepant to treat acute migraines, and it is also being studied for migraine prophylaxis [5].

Our case series presents three cases of post-traumatic CH. These patients were provided a sample of rimegepant. All three patients reported a varying degree of decrease in the intensity of headaches but no effect on the frequency of headaches. It has been demonstrated that CH alone is not associated with higher serum levels of CGRP. In CH patients, an increase in CGRP due to nociceptive input from the cervical spine's degenerative alterations may promote peripheral sensitization, which in turn may bring on a new case of a primary headache disorder with a cervicogenic trigger or worsen an existing primary headache disorder [15]. This may explain why rimegepant caused a decrease in headache intensity in these patients.

**Figure 1:**
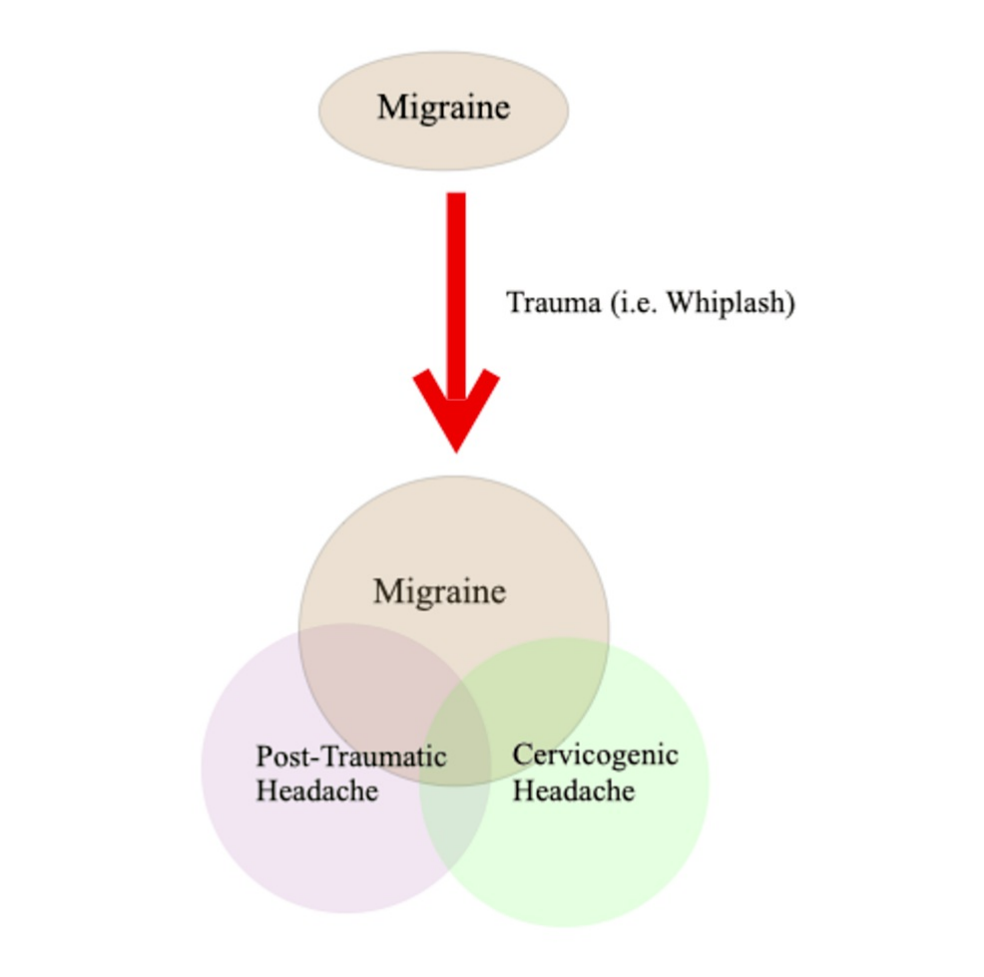
Potential outcome of a patient with a history of migraines who suffered trauma secondary to whiplash.

Post-traumatic headache (PTH) is the most frequent symptom after mild traumatic brain injury (mTBI) and results in an increased release of CGRP [16,17]. After trauma, it is challenging to determine if the headaches are related to the cervical spine or the brain, as traumas may cause persistent neck pain and mTBI. A history of migraine is also a risk factor for persistent headache symptoms after trauma [8]. This further increases the challenge of determining the root cause of the headaches, as many of these headaches share very similar presentations and can overlap with one another (Figure 1).

Our study includes limitations as we solely relied on the patient's history, physical examination, and imaging to make a clinical diagnosis. While a nerve block of the cervical spine is not necessary to diagnose CH, it was not performed to confirm the diagnosis further. The patients may only have PTH after the MVA or are experiencing both CH and PTH. Since the patients knew they were taking a new medication, psychological factors may have also contributed to the pain relief.

## Conclusions

Our study presents the use of rimegepant in three cases of post-traumatic CH, and the patients reported a decrease in the intensity of their headaches. However, the study has limitations, including a small sample size and psychological factors that may have also contributed to pain relief. An elevated CGRP has not been proven to be associated with CH; however, rimegepant's impact on headache intensity suggests a connection between the two. Further research with larger sample sizes and controlled studies is needed to confirm the effectiveness of rimegepant in treating CH and determine its effect on other symptoms. It is also important to note that CH is a complex condition that can be challenging to diagnose, especially in the context of post-traumatic headaches, and may require a multidisciplinary approach to manage.
